# Exploratory Cohort Study of Depressive Symptoms in South Africans with HIV-1 Subtype C: Associations with Kynurenine Pathway Metabolites and Inflammatory Markers

**DOI:** 10.1007/s11481-025-10278-3

**Published:** 2026-01-23

**Authors:** Monray Edward Williams, Lusilda Schutte, Levanco K. Asia, Marié P. Wissing, Esmé Jansen van Vuren

**Affiliations:** 1 Biomedical and Molecular Metabolism Research (BioMMet), North-West University, Potchefstroom, South Africa; 2 Africa Unit for Transdisciplinary Health Research (AUTHeR), North-West University, Potchefstroom, South Africa; 3 Hypertension in Africa Research Team (HART), North-West University, Potchefstroom, South Africa; 4 South African Medical Research Council Unit for Hypertension and Cardiovascular Disease, North-West University, Potchefstroom, South Africa

**Keywords:** HIV-1 subtype C, Depression risk, Tryptophan–kynurenine pathway, Quinolinic acid (QUIN), Immune–metabolic dysregulation

## Abstract

Background: Depression is common in people living with HIV and may involve immune–metabolic dysregulation. Altered tryptophan (Trp) metabolism via the kynurenine (Kyn) pathway, particularly quinolinic acid (QUIN), has been linked to neuroinflammation and depression, yet data from South Africans with HIV-1 subtype C are limited. Methods: In this exploratory cohort, treatment-naïve adults with HIV were assessed at baseline in 2010 (n = 69) and followed up in 2015 (n = 40). Targeted LC-MS/MS quantified Trp–Kyn metabolites (Trp, Kyn, KA, QUIN) and ELISAs measured immune markers (suPAR, IL-6, hsCRP, sCD163, NGAL). Depressive symptoms were evaluated using the PHQ-9 at baseline and follow-up. Results: Baseline QUIN was associated with the PHQ-9 total score (adjusted R²=0.245; β=0.417; *p *= 0.004, *p*(Hochberg) = 0.044) and also increased odds of depression risk (OR=61.1; 95%CI 2.24–1664.76; *p *= 0.015, *p*(Hochberg) = 0.030) at baseline. Baseline NGAL was significantly lower in the follow-up group with self-harm thoughts compared to the group with no–self-harm thoughts (*p* = 0.008) and lower NGAL predicted greater odds of follow-up self-harm thoughts (OR = 0.007, 95% CI: <0.001–0.572, *p *= 0.027, *p*(Hochberg) = 0.027). When PHQ-9 subscales were examined, baseline QUIN, Kyn, hsCRP, and suPAR showed positive associations with the baseline somatic subscale (β range= 0.34–0.57, all *p* < 0.01), whereas the baseline cognitive–affective subscale was not associated with any marker. No baseline markers were associated with follow-up depression risk or scores. Over five years, suPAR decreased whereas hsCRP did not change significantly, however changes in these markers over time did not reveal any associations with the PHQ-9 total or subscale scores. Conclusions: In this exploratory cohort, higher peripheral QUIN may be linked to depression risk, particularly with somatic rather than cognitive–affective symptom dimensions. Lower baseline NGAL was the only marker associated with later self-harm thoughts, suggesting a potential neuroimmune signal that warrants further investigation. Larger, adequately powered longitudinal studies with repeated metabolomic assessments are warranted to confirm this finding and clarify temporal and causal relationships between immune–metabolic dysregulation and specific depressive symptom profiles.

**Graphical Abstract:**

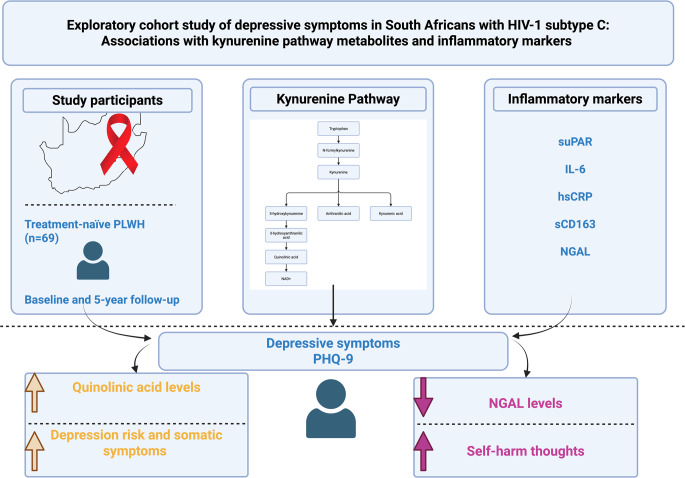

## Introduction

HIV remains a global health concern despite 40 years of HIV-1 research (UNAIDS 2025). Currently, 38 million people are living with HIV (UNAIDS 2025). While combination antiretroviral therapy (cART) has reduced the viral replication of the virus, neurological complications seem to persist, including the development of HIV-associated neurocognitive disorders (Bandera et al. 2019; Williams et al. 2020a) and HIV-related depression (Carrico et al. 2022).

Depression is a prevalent comorbidity among people with HIV, with significant implications for disease progression, treatment adherence, and overall quality of life (Carrico et al. 2022). The aetiology of HIV-associated depression is multifactorial, with evidence pointing to both immune activation and metabolic dysregulation as key contributors (Rivera-Rivera et al. 2016). Notably, chronic inflammation and alterations in the tryptophan (Trp)-kynurenine (Kyn) metabolic pathway have been implicated in the pathogenesis of depression within people with HIV (Drivsholm et al. 2021). However, there is limited research on these associations in South African cohorts, particularly in people with HIV-1 subtype C, which is the predominant strain in the region.

The Trp-Kyn pathway plays a crucial role in neuroimmune interactions and has been extensively studied in relation to neuroinflammation and neurodegeneration. Trp is catabolized by the enzyme indoleamine 2,3-dioxygenase (IDO) into Kyn, which is further metabolized into neuroactive metabolites such as kynurenic acid (KA) and quinolinic acid (QUIN) (Rivera-Rivera et al. 2016). QUIN has been implicated in excitotoxicity and neuroinflammation, both of which are thought to contribute to depressive symptoms in people with HIV (Mudra Rakshasa-Loots et al. 2022). Moreover, immune markers such as soluble urokinase plasminogen activator receptor (suPAR), interleukin-6 (IL-6), high-sensitivity C-reactive protein (hsCRP), soluble CD163 (sCD163), and neutrophil gelatinase-associated lipocalin (NGAL) are associated with systemic inflammation and have been suggested as potential markers for depression risk in various populations (Mudra Rakshasa-Loots 2023).

Increasing evidence indicates that these same inflammatory mediators are closely linked to alterations in Trp–Kyn metabolism, a pathway that connects immune activation with neuroactive metabolite production. IL-6 engages the Janus kinase/signal transducer and activator of transcription 3 axis to upregulate IDO1, thereby shifting Trp metabolism toward Kyn production (Mondanelli et al. 2021). Macrophage activation (reflected by sCD163) is associated with elevated IDO1 pathway activity (higher Kyn/Trp), suggesting that monocyte/macrophage inflammation may be one source of enhanced Trp catabolism (Shorer et al. 2024). In patients with chronic renal failure, elevated suPAR was significantly associated with higher Kyn and lower Trp levels (i.e., increased Trp to Kyn catabolism) compared to controls. This suggests that suPAR (reflecting immune activation/inflammation) may track Trp metabolism changes, although mechanistic steps are less well detailed (Pawlak et al. 2010). NGAL, a marker of neutrophil activation and systemic inflammation, has also been examined in relation to Trp–Kyn metabolism. Although NGAL reflects innate immune and oxidative stress pathways, direct mechanistic links to IDO1 or Tryptophan 2,3-dioxygenase (TDO2) activation remain unclear. In a cohort of treatment-naïve people living with HIV, NGAL was measured alongside Trp–Kyn metabolites, suggesting a possible association between innate immune activation and enhanced Trp catabolism, though causality has not been demonstrated (Sultana et al. 2023; Williams et al. 2025a).

Despite growing interest in the role of immune-metabolic interactions in HIV-associated depression, studies investigating the relationship between immune markers, Trp-Kyn metabolites, and depression risk in South African people with HIV, particularly those who are treatment-naïve, remain scarce.

Although South Africa has made progress toward the 2025 Joint United Nations Programme on HIV/AIDS (UNAIDS) goal of 95% awareness of HIV status (Frescura et al. 2022), achieving the target of 95% cART coverage remains unlikely – especially in rural areas. According to the 2022 Sixth South African National HIV Prevalence, Incidence, and Behaviour Survey (SABSSM VI), 90% of people living with HIV aged 15 years and older were aware of their status, 91% of those aware were on cART, and 94% of those on cART were virally suppressed, reflecting significant progress but also ongoing disparities in treatment coverage, particularly in underserved areas (Ramlagan 2023). During 2010–2015, national cART rollout was ongoing and heterogeneous across provinces, with substantial variability in treatment coverage, guideline implementation, and health-system capacity (Bor et al. 2021). The UNAIDS Global AIDS Update documented uneven cART access and ongoing gaps in viral suppression across sub-Saharan Africa during this period (UNAIDS 2025). These disparities were reflected in marked regional and urban–rural differences in cART uptake and HIV prevalence within South Africa (Gibbs et al. 2020; Ugwu and Ncayiyana 2022).

This further highlights the importance of investigating the clinical implications of HIV-related inflammation in a treatment-naïve cohort. Examining treatment-naïve individuals at baseline allows for assessment of immune, metabolic, and depressive-symptom relationships before cART-related immune reconstitution or suppression of inflammatory pathways occurs. Prior longitudinal research has demonstrated that initiating cART substantially changes circulating inflammatory and metabolic markers, which can confound interpretation of baseline associations (Angelidou et al. 2018; Funderburg et al. 2024). This underscores the scientific value of focusing on an untreated cohort to clarify early neuroimmune–metabolic mechanisms that may contribute to depression risk in HIV-1 subtype C infection.

South Africa provides a particularly distinctive setting for investigating underlying neuroimmune mechanisms in the context of HIV-1 subtype C infection, as it remains limited in investigation in comparison to the subtype B that is largely present in the Global North (Williams et al. 2025b). Subtype C, predominant in Southern Africa, shows sequence/functional features in viral proteins (e.g., Transactivator of Transcription (Tat), Viral protein R(Vpr)) with potential neuroimmune consequences that may differ from subtype B. Emerging work suggests that subtype C variation results in amino acid changes in viral protein including but not limited to Tat and Vpr and these can modulate neurotoxicity, monocyte/macrophage activation, and IDO1-driven Kyn flux, thereby shifting the KA/QUIN balance toward neurotoxic tone in inflammatory contexts (Campbell et al. 2007; Samikkannu et al. 2009; Williams et al. 2020b; Asia et al. 2024a, b).

Understanding these relationships could provide critical insights into the biological mechanisms underlying depression in this population, considering the high prevalence, and inform targeted interventions. While neuroinflammation and neurometabolism occur in the brain, investigating these processes in the blood offers a non-invasive way to explore potential markers.

This study aims to explore the relationship between peripheral immune markers, Trp-Kyn metabolites, and depression symptoms in a cohort of South Africans living with HIV, who were treatment-naïve at baseline and followed up over a 5-year period. We hypothesise that increased levels of inflammatory markers and neurotoxic Trp-Kyn metabolites, such as QUIN, will be associated with a higher risk of depression. By addressing these research gaps, our findings may contribute to a more comprehensive understanding of the biological underpinnings of HIV-associated depression and inform future therapeutic strategies.

## Methodology

### Study Participants

**Fig. 1:**
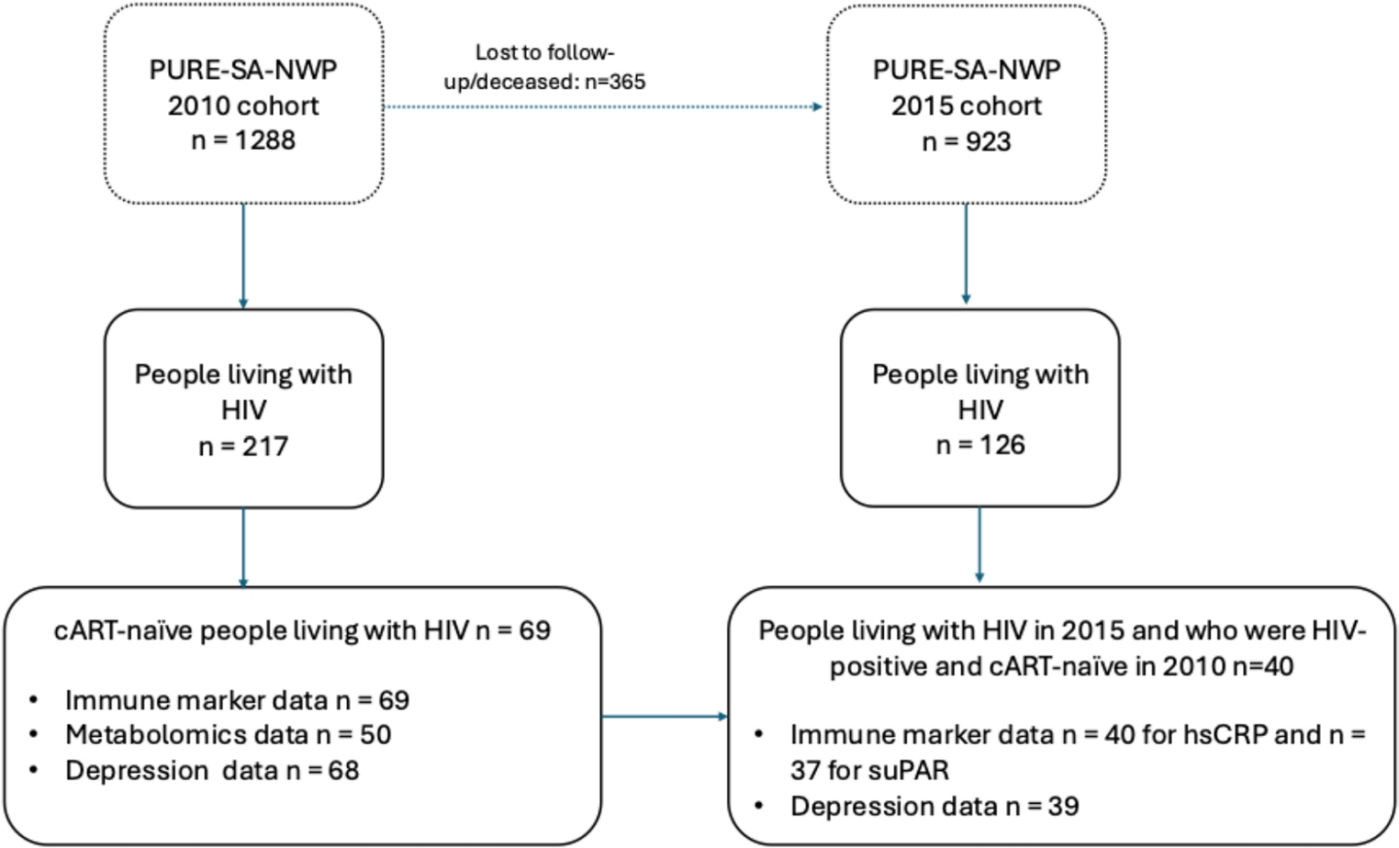
Overview of the PURE-SA-NWP cohort and the sub-cohort used for the current study. The flow diagram illustrates participant follow-up from baseline (2010) to follow-up (2015), focusing on treatment-naïve individuals living with HIV at baseline. Data availability is shown for immune markers (hsCRP and suPAR), metabolomics, and depression assessments at follow-up

Participants in this study were drawn from the South Africa – North West province (NWP) leg of the multi-country Prospective Urban and Rural Epidemiology (PURE-SA-NWP) study (Teo et al. 2009). For PURE-SA-NWP, men and women between 35 and 70 years old of African descent were recruited from urban and rural areas in the North West province, South Africa. The first wave of data collection in PURE-SA-NWP took place in 2005 from *n* = 2,010 participants, with the second wave in 2010 from *n* = 1,288 participants and the third wave in 2015 from *n* = 923 participants. For the purpose of the current study, the 2010 wave was used as baseline and the 2015 wave as follow-up. In 2010, *n* = 69 participants who tested positive for HIV-1 and were treatment-naive at the time of data collection formed the sample for this study. Baseline immune marker data were available for all participants (*n* = 69/69; 100%). However, targeted metabolomics data were available for only 50 participants at baseline (*n* = 50/69; 72%) due to limited sample volume. Specifically, not all participants who provided blood samples at baseline had sufficient remaining serum or plasma for metabolomic assays after immune marker analyses were completed. Consequently, metabolomic analyses were conducted on this subsample. Baseline depression data (Patient Health Questionnaire-9 [PHQ-9]) were available for *n* = 68/69 (99%) participants (Fig. 1).

A total of *n* = 40 participants (58%) were retained at follow-up. Of these, *n* = 8 participants remained treatment-naïve and *n* = 32 participants were receiving cART. The use of cART in 2015 was accounted for in statistical analyses. At follow-up, hsCRP data were available for *n* = 40/40 (100%), suPAR for *n* = 37/40 (93%), and PHQ-9 data for *n* = 39/40 (98%). These datasets did not completely overlap: 38 participants had both hsCRP and depression data, while all participants with suPAR data (*n* = 37) also had depression data. No metabolomics data were collected at follow-up. A detailed breakdown of participant inclusion and data overlap is shown in Fig. 1; Table 1. Attrition from baseline (*n* = 69) to follow-up (*n* = 40; 58%) may have introduced selection bias if loss to follow-up was non-random (e.g., differential mortality, migration, or ART initiation). Because targeted metabolomics data were available for only 50 participants, the statistical power for those analyses was limited, and the resulting confidence intervals should be interpreted with caution.

**Table 1:** Study characteristics for depression risk groups and the overall cohort at baseline 2010

Study characteristics of full cohort at baseline	At risk for depression (*n* = 36)	Not at risk for depression (*n* = 32)	Overall cohort (*n* = 69)	*n* (available)
Age in years, mean (SD)	47.1 (6.2)	47.8 (7.8)	47.3 (6.9)	69/69 (100%)
Sex, female n (%)	29 (81)	20 (63)	49 (71)	69/69 (100%)
^a^CD4^+^ cells/mm^3^, mean (SD)	311.2 (155.3)	282.6 (142.7)	296.1 (146.2)	41/69 (59.4%)
Locality, rural n (%)	15 (42)	19 (59)	34 (49)	69/69 (100%)
^b^Employment status, employed n (%)	6 (18)	4 (13)	10 (16)	64/69 (92.8%)
^c^Level of education, no formal education, n (%)	11 (31)	7 (22)	18 (27)	67/69 (97.1%)
^d^Smoking status, former and/or current use (%)	18 (50)	22 (69)	41 (59.4)	67/69 (97.1%)
^e^Alcohol consumption, currently consuming any alcohol product n (%)	12 (33)	15 (47)	28 (40.6)	67/69 (97.1%)
Body mass index, mean (SD)	23.3 (6.2)	23.6 (5.2)	23.4. (5.7)	69/69 (100%)
IL-6, pg/mL, median (IQR)	5.97 (2.86–11.92)	4.37 (2.88–7.11)	5.3 (2.9–7.9)	69/69 (100%)
hsCRP, mg/L, median (IQR)	3.72 (1.54–15.60)	2.01 (0.96–5.07)	2.82 (1.2–8.3)	69/69 (100%)
suPAR, ng/mL, mean (SD)	4.41 (1.7)	4.11 (1.8)	4.28 (1.7)	69/69 (100%)
NGAL, ng/mL, mean (SD)	54.9 (23.1)	48.9 (17.0)	52.1 (20.4)	69/69 (100%)
sCD163, ng/mL, mean (SD)	771.0 (356.1)	770.3 (374.6)	775.9 (362.1)	69/69 (100%)
Study characteristics of subsample with metabolomics data	At risk for depression (*n* = 28)	Not at risk for depression (*n* = 22)	Overall sub-cohort (*n* = 50)	
Trp, 𝜇g/mL, mean (SD)	14.84 (7.2)	16 (6.0)	15.35 (6.70)	50/69 (72.5%)
Kyn, 𝜇g/mL, mean (SD)	0.88 (0.2)	0.86 (0.3)	0.87 (0.3)	50/69 (72.5%)
Kyn/Trp ratio, mean (SD)	0.07 (0.03)	0.06 (0.04)	0.07(0.03)	50/69 (72.5%)
QUIN, 𝜇g/mL, median (IQR)	0.15 (0.09–0.24)	0.08 (0.05–0.13)	0.11 (0.07–0.18)	50/69 (72.5%)
KA, ng/mL, median (IQR)	0.71 (0.32–1.35)	1.12 (0.7–2.19)	0.95 (0.005–1.99)	50/69 (72.5%)
KA/QUIN ratio, median (IQR)	0.005 (0.002–0.01)	0.02 (0.006–0.03)	0.008 (0.003–0.020	50/69 (72.5%)

^a^CD4^+^ count data were available for *n* = 20 participants in the depression-risk group, *n* = 20 participants in the not-at-risk-for-depression group, and *n* = 41 participants in the overall cohort

^b^Employment data were missing for *n* = 5 participants

^c^Level of education, ^d^Smoking status, ^e^alcohol consumption data were missing for 2 participants

Skewed variables (IL-6, hsCRP, QUIN, KA and KA/QUIN ratio) are presented as median (IQR). All skewed variables were presented as untransformed for presentation purposes. Across the full cohort of 69 participants, data completeness varied by measure. Baseline depression symptom data were available for 68 of 69 participants (98.6%), while targeted metabolomic assays were available for 50 of 69 participants (72.5%) due to limited sample volume. At follow-up, hsCRP values were available for 40 of 40 participants (100%), suPAR for 37 of 40 (92.5%), and PHQ-9 scores for 39 of 40 (97.5%)

HIV status and CD4^+^ count were determined as outlined in previous studies (Asia et al. 2024a, b). Viral load (VL) was not collected at baseline nor follow-up because the parent study focused on cardiovascular outcomes (Botha et al. 2014). As all participants were treatment-naïve at baseline, VL was expected to be detectable/high. In prior work using this cohort we successfully sequenced HIV-1 genes from plasma (Williams 2024; Williams et al. 2024), which is consistent with detectable viremia; however, this does not replace measured VL. The absence of VL measurements is further acknowledged in the limitations section. The study protocol received approval from the Health Research Ethics Committee of the North-West University in South Africa (NWU-00016-10-A1 for PURE-SA-NWP; NWU-00002–07-A2 for FORT3 of which the depression data in PURE-SA-NWP formed part; and NWU-00106-22-A1 for the PURE-SA-NWP sub study on immune/metabolic profiles of treatment-naïve HIV positive participants in the 2010 wave).

### Sociodemographic and Anthropometric Variables and Depression Symptoms

Trained fieldworkers assisted participants in completing questionnaires in their home language. A questionnaire from the international PURE study was used to gather demographic and lifestyle data, including age, sex, locality (categorized as urban or rural), current employment status, education level (categorized as no formal education vs. primary to university), alcohol consumption (categorized as never use vs. current use of any alcohol product), and smoking status (categorized as no use vs. former and/or current use). Anthropometric measurements were conducted following the guidelines of the International Society for the Advancement of Kinanthropometry. These included body height (measured using an Invicta Stadiometer, IP 1465, Invicta, London, UK), body weight (measured with a Precision Health Scale, A&D Company, Tokyo, Japan), and waist circumference (measured using a Holtain unstretchable metal tape). Body mass index (BMI) was calculated as weight in kilograms divided by the square of height in meters (kg/m²).

### Depression Symptoms

Depression symptoms were assessed using the nine-item Patient Health Questionnaire-9 (PHQ-9), a widely validated screening tool for assessing the presence and severity of depressive symptoms according to the fourth edition of the Diagnostic and Statistical Manual of Mental Disorders (DSM-IV) (Kroenke et al. 2001). The PHQ-9 was included as an additional measure in the PURE-SA-NWP study and did not form part of the global PURE study. The PHQ-9 evaluates the frequency of nine symptoms of a major depressive episode over the preceding two weeks by using a Likert scale ranging from 0 (‘not at all’) to 3 (‘nearly every day’). Participants’ responses were summed to generate a total score (ranging from 0 to 27) with higher scores representing higher depressive symptom severity. Participants were categorised into two groups based on their total PHQ-9 score: the presence of none/minimal to mild depressive symptoms (PHQ-9 total score < 10) classified individuals not at risk for depression while the presence of moderate-to-severe depressive symptoms (PHQ-9 total score ≥ 10) classified those at risk for depression (Kroenke et al. 2001). In addition to the total PHQ-9 score, we derived two subscale scores to examine potential symptom-domain–specific biological associations as done previously (Naudé et al. 2025). Following established two-factor models of the PHQ-9, we calculated a cognitive-affective subscale (items 1, 2, 6–9) and a somatic subscale (items 3–5) (Kroenke et al. 2001; Patel et al. 2019). Both subscale scores were treated as continuous variables and analysed in multivariable regression models assessing associations with immune and metabolite markers. Internal consistency of the PHQ-9 in this cohort was assessed using Cronbach’s alpha and McDonald’s omega (SPSS version 30), first for the total scale and then for the cognitive-affective and somatic subscales separately. Alpha and omega were computed separately for each wave (2010 baseline; 2015 follow-up). Lastly, we examined item 9 of the PHQ-9, which assesses self-harm thoughts in relation to relevant immune markers and metabolites. Scores on the item were recoded to create a binary variable that was used for analyses: endorsing any self-harm thoughts (item 9 ≥ 1) was compared to those without such thoughts (item 9 = 0).

### Targeted Metabolomics Analysis and LC-MS/MS Analyses

Details of all samples, chemicals, and standards used for metabolomic analysis were previously outlined in our primary study (Asia et al. 2024b). We examined the metabolic profiles of Trp, Kyn, KA, and QUIN, as they may contribute to the pathogenesis of HIV. In summary, high-performance liquid chromatography (HPLC) was employed for initial profiling, while targeted quantification was conducted using liquid chromatography–tandem mass spectrometry (LC-MS/MS). The analysis utilised an Agilent 1200 series HPLC system coupled with a 6470 triple quadrupole mass spectrometer (Agilent Technologies) as per the methods described in (Asia et al. 2024b). In addition, the Kyn/Trp ratio was calculated as an index of IDO activity (Badawy and Guillemin 2019), whereas the KA/QUIN ratio was derived as a measure of neuroprotective versus neurotoxic balance within the pathway (Parrott et al. 2016b).

### Immune Marker Analysis

Immune marker analysis in blood samples was conducted using the preparation method outlined in our primary study (Asia et al. 2024b). The immune markers assessed included suPAR, IL-6, hsCRP, sCD163, and NGAL, selected for their relevance to HIV-1 pathogenesis. All tests demonstrated acceptable precision, with intra-assay coefficients of variation below 8% and inter-assay coefficients below 10%.

### Statistical Analysis

Statistical analysis was carried out using SPSS (version 30, IBM, USA), with p-values less than 0.05 deemed statistically significant. Normality of continuous variables was evaluated using quantile–quantile (Q–Q) plots and skewness and kurtosis z-scores (statistic divided by its standard error), following the criterion of |z| > 3.29 to indicate significant deviation from normality for medium-sized samples (50 < *n* < 300) (Kim 2013). Variables exceeding this threshold (hsCRP (2010 and 2015), IL-6, QUIN, KA and KA/QUIN) were log-transformed prior to analysis. This procedure provided an objective, sample-size-appropriate criterion and reduced over-reliance on omnibus normality tests that can be overly sensitive in larger samples. After transformation, all variables demonstrated acceptable skewness and kurtosis values |z| < 3.29 (Kim 2013).

Analyses were conducted on transformed values, but descriptive statistics are presented on untransformed data for interpretability. To test for group differences at baseline, specifically for sex, locality, employment status, level of education, smoking, and alcohol consumption between depression risk groups, χ2 tests were used. To determine if there were differences in potential continuous covariates (age, CD4^+^ counts and BMI), the Mann-Whitney U test was used.

To identify potential sociodemographic and lifestyle covariates associated with marker levels, we conducted penalized regression analyses using the least absolute shrinkage and selection operator (LASSO) implemented in IBM SPSS Statistics (version 30). Separate models were fitted for each marker (suPAR, NGAL, sCD163, hsCRP, IL-6, Kyn, Trp, Kyn/Trp ratio, KA, QUIN and KA/QUIN ratio). The following candidate covariates were included in all models: age, sex, locality (urban/rural), education level, employment status, body mass index (BMI), tobacco use, and alcohol consumption. Predictors were automatically standardized prior to estimation to ensure comparability of penalization across variables. Models were fitted using five-fold cross-validation with an α-penalty of 1.0 (LASSO), and model performance was evaluated using training, test, and holdout R² values. Variables with regression coefficients shrunk to zero were interpreted as not contributing meaningfully to marker variation. This approach was employed as a data-driven sensitivity analysis to complement the a priori covariate selection used in the main regression analyses. For all follow-up analysis, only cART status was included in the model, as BMI, smoking and alcohol use did not differ significantly over time.

To assess the associations between depression (at baseline and follow-up) and immune and metabolic markers, both categorised (risk/not at risk) and continuous versions of the depression variable (PHQ-9 total and subscale scores) were used. For the categorical version of the depression variable, the Mann Whitney U tests were conducted to compare mean marker levels between groups. Then, analysis of covariance (ANCOVA) was conducted with levels of immune markers/metabolites as the dependent variables to compare the immune marker/metabolite levels between risk and not at risk groups while adjusting for confounders. For significant findings reported in ANOCOVA for categorial variables, logistic regression analyses were conducted to determine the odds of baseline immune and metabolic markers to increase the likelihood of depression risk at baseline and follow-up (i.e., categorical outcome), adjusting for confounders.

For the continuous version of the depression variable (PHQ-9 total score as well as subscale scores), multiple regression analysis was conducted using the enter method to determine relationships between baseline immune/metabolic markers and both baseline and follow-up PHQ-9 total and subscale scores, adjusting for confounders. All tests were two-sided with α = 0.05. To control for multiple comparisons across the 11 immune/metabolic markers, the Hochberg step-up procedure was applied to adjust *p*-values (family-wise error rate, FWER). We report both the nominal *p*-value (*p*) and the adjusted *p*-value (p(Hochberg)), and results were considered statistically significant at p(Hochberg) < 0.05.

We also collected follow-up immune marker data for hsCRP (*n* = 38) and suPAR (*n* = 37) with corresponding depression data and aimed to determine whether changes in inflammation over time were associated with depression risk (categorical outcome) and the PHQ-9 total and subscale scores (continuous outcomes) at follow-up using logistic regression and multiple regression analyses, respectively. Percentage change was calculated as %Δ = [(Follow up-Baseline)/Baseline] × 100.

## Results

### Study Characteristics

This study included a total of *n* = 69 people living with HIV, all of whom had subtype C and were treatment-naïve at baseline. The overall cohort had a mean age of 47.3 (±6.9) years at baseline, with females comprising the majority (71%) of participants. Additionally, only a subset of participants (*n* = 41/69, 59%) had CD4^+^ count data, with a mean of 296.1 (± 146.2) cells/mm³ (Table 1). Depression data were available for *n* = 68/69, 99% people living with HIV (one participant missing PHQ-9 data), of whom *n* = 36 were at risk and *n* = 32 not at risk for depression at baseline. There were no significant differences between participants at risk for depression and those not at risk in terms of CD4⁺ counts or other study characteristics, including age, sex, locality, employment status, level of education, smoking, and alcohol consumption (*p* > 0.05). Due to limited sample availability, metabolomics analysis was only conducted for *n* = 50/69, 72% people living with HIV at baseline (Table 1). The PHQ-9, as well as its subscales, showed acceptable internal consistency reliability (Tavakol and Dennick 2011): For the total scale, α = 0.823 and ω = 0.819 at baseline and α = 0.749 and ω = 0.748 at follow-up. For the cognitive-affective subscale, α = 0.787 and ω = 0.788 at baseline and α = 0.632 and ω = 0.612 at follow-up. For the somatic subscale, α =.641and ω = 0.660 at baseline and α = 0.555 and ω = 0.695 at follow-up.

Penalized regression analyses using the LASSO method were performed to identify potential sociodemographic and lifestyle covariates associated with baseline marker levels. Across all markers tested, including suPAR, NGAL, sCD163, hsCRP, IL-6, Kyn, Trp, Kyn/Trp ratio, KA, QUIN and KA/QUIN ratio, the model coefficients for all predictors were shrunk to zero, indicating no substantial independent associations with the markers after penalization. For NGAL and sCD163, LASSO retained some predictors, but test/holdout $\:{\mathrm{R}}^{2}$were negative (e.g., NGAL: test $\:{\mathrm{R}}^{2}=-0.66$, holdout $\:{\mathrm{R}}^{2}=-0.21$; sCD163: test $\:{\mathrm{R}}^{2}=-0.74$, holdout $\:{\mathrm{R}}^{2}=-0.79$), indicating no generalizable predictive signal. These results suggest that none of the tested demographic or lifestyle variables (age, sex, locality, education, employment, BMI, tobacco use, or alcohol consumption) demonstrated stable or meaningful predictive relationships with marker levels in this cohort. Given that the LASSO regression analyses did not identify any stable demographic or lifestyle predictors of marker variability, covariate selection for all subsequent analyses was guided by theoretical and empirical considerations rather than automated model selection. To avoid model overfitting and ensure comparability with prior work, all subsequent ANCOVA and regression analyses were adjusted for body mass index (BMI), education, sex, and locality, which were identified a priori as common confounders in this context. BMI was included given its known mediating/moderating role in the association between circulating inflammatory markers (such as CRP, IL-6) and depressive symptoms (Howren et al. 2009). Education was retained as a proxy for socioeconomic position, influencing both lifestyle behaviours and psychosocial exposures linked to immune and metabolic regulation (Matthews and Gallo 2011). Sex was included because of well-documented sexual dimorphism in immune–metabolic responses and mental health outcomes: females are more vulnerable to the depressogenic effects of inflammation than males, and immune–stress coupling differs by sex (Bekhbat and Neigh 2018). Locality (urban/rural) was included a priori as a design variable because the PURE-SA-NWP sampling frame was stratified by urban and rural communities. Locality represents a contextual factor that plausibly influences both exposures (e.g., infectious burden, healthcare access, nutritional diversity, and environmental stressors) and outcomes such as depressive symptoms through differences in service availability, economic opportunity, and social environment (Teo et al. 2009). Previous analyses within the same cohort examining inflammatory and metabolic markers have demonstrated systematic urban–rural differences and treated locality as an important confounder (Jansen van Vuren et al., 2025; Williams et al. 2025a). Accordingly, locality was retained in all adjusted models irrespective of statistical significance to align with the study’s stratified design and prior analyses.

### Comparison of Baseline Immune Marker/Metabolite Levels between Depression Risk Groups (Categorical) at Baseline and Follow-up

We conducted Mann–Whitney U tests to compare baseline immune and metabolic marker levels between baseline depression risk groups (2010) as shown in Table 1. Among all markers, KA/QUIN ratio was lower (*p* = 0.007) while QUIN was higher (*p* = 0.010) in the at risk for depression group when compared to the not at risk group. However, after applying the Hochberg step-up procedure to control for multiple testing across 11 markers, neither remained statistically significant (*p*(Hochberg)_KA/QUIN = 0.077; *p*(Hochberg)_QUIN = 0.100). No other baseline markers differed significantly between baseline depression risk groups (all *p*(Hochberg) ≥ 0.513).

After adjusting for sex, locality, education level, and BMI, baseline KA/QUIN levels were lower in the at-risk group (0.015, 95% CI [0.005, 0.024]) compared with the not-at-risk group (0.021, 95% CI [0.010, 0.033]; *p* = 0.076). In contrast, baseline QUIN levels were higher in the at-risk group (0.002, 95% CI [0.001, 0.002]) than in the not-at-risk group (0.001, 95% CI [0.001, 0.001]; *p* = 0.013). However, none of these differences remained statistically significant after Hochberg correction for multiple testing (p(Hochberg)_QUIN = 0.143; *p*(Hochberg)_KA/QUIN = 0.836). No other baseline markers differed between the groups (all *p*(Hochberg) ≥ 0.941).

We also conducted Mann-Whitney U tests to compare baseline immune and metabolic marker levels between follow-up depression risk groups. At follow-up, none of the baseline markers differed between depression risk groups after correction (all *p*(Hochberg) ≥ 0.704). The lowest nominal *p*-value was observed for sCD163 (*p* = 0.064), which was not significant after Hochberg adjustment (*p*(Hochberg) = 0.704). Further, after adjusting for sex, locality, education level, and BMI in an ANCOVA, no baseline immune or metabolic markers differed significantly between the follow-up depression risk groups after correction for multiple testing using the Hochberg step-up procedure (all *p*(Hochberg) ≥ 0.984).

To explore whether immune or metabolic dysregulation differed by thoughts of self-harm specifically, we examined the baseline and follow-up PHQ-9 self-harm item (item 9) in relation to baseline marker levels. Participants with any baseline self-harm thoughts (item 9 ≥ 1) did not significantly differ from those without self-harm thoughts (item 9 = 0) in any baseline marker both prior and after adjustment for multiple comparisons using Mann-Whitney U tests. Further, no significant differences were observed across all baseline markers between the baseline PHQ-9 self-harm item categories after covariate adjustment in ANCOVA (all *p* ≥ 0.256; Hochberg-adjusted *p* > 0.05).

Similarly, no baseline marker differed between follow-up groups with self-harm thoughts (item 9 ≥ 1) and those without self-harm thoughts (item 9 = 0), either before or after adjustment for multiple comparisons using Mann–Whitney U tests. After adjusting for BMI, locality, gender, and education, no baseline markers differed between the follow-up self-harm and no–self-harm groups except NGAL, which was significantly lower in the self-harm group (mean = 31,934 ng/mL, 95% CI [17,145, 46,723]) compared with the no–self-harm group (mean = 55,450 ng/mL, 95% CI [48,253, 62,646]; *p* = 0.008, *p*(Hochberg) = 0.008). All other baseline markers showed no significant between-group differences (all adjusted *p*-values > 0.05).

### Associations between Baseline Immune Marker/Metabolite Levels with Depression Risk (Categorical) at Baseline and Follow-up with Significant Findings

Given that QUIN and KA/QUIN ratio were the only baseline markers nominally different in the at-risk group (see section "Comparison of Baseline Immune Marker/Metabolite Levels between Depression Risk Groups (Categorical) at Baseline and Follow-up"), we ran logistic regressions only for these markers (adjusted for sex, locality, education, and BMI). Higher baseline QUIN was associated with greater odds for baseline depression-risk (OR = 61.1, 95% CI: 2.24–1664.76, *p* = 0.015; *p*(Hochberg) = 0.030), whereas the KA/QUIN ratio was not (odds ratio [OR] = 0.559, 95% confidence interval [CI]: 0.301–1.038, *p* = 0.066; *p*(Hochberg) = 0.066). Further, considering that baseline NGAL was the only marker significantly lower in the follow-up group with self-harm thoughts, we ran a logistic regression (adjusted for sex, locality, education, and BMI). Lower baseline NGAL was associated with greater odds of reporting self-harm thoughts at follow-up (OR = 0.007, 95% CI: <0.001–0.572, *p* = 0.027) *p*(Hochberg) = 0.027). Given the small sample, wide confidence intervals, and multiple testing, this estimate should be interpreted cautiously as hypothesis-generating.

### Relationship between Baseline Immune/Metabolite and Baseline/Follow-up Depression Total Scores (Continuous)

Separate adjusted multivariate regression analyses were conducted to examine the relationships between baseline immune and metabolite markers and baseline and follow-up depression scores (PHQ-9 total score) in the overall cohort. Models were adjusted for sex, locality, education level, and BMI in baseline analyses, with cART use additionally included as a covariate in follow-up analyses. Across the 11 baseline markers, only QUIN showed a significant positive association with the PHQ-9 total scores at baseline (β = 0.426, adjusted R² = 0.229, *p* = 0.004). This association remained statistically significant after correction for multiple testing using Hochberg’s step-up procedure (*p*(Hochberg) = 0.044 < 0.05). No baseline markers were associated with follow-up PHQ-9 total scores after correction for multiple testing using Hochberg’s step-up procedure (all *p*(Hochberg) ≥ 0.710).

To explore whether associations were specific to specific depression risk groups, we conducted separate multivariate regression analyses within the at risk and not at risk groups (risk group established at baseline). Within the at risk group, we fit separate multivariable regressions of each baseline marker on baseline PHQ-9 total score (adjusted for sex, locality, education, and BMI). The PHQ-9 total score showed a positive association with baseline sCD163 (β = 0.448, *p* = 0.028) and a trend with the Kyn/Trp ratio (β = 0.456, *p* = 0.055), but neither survived multiple-testing control using the Hochberg step-up procedure across 11 markers (all adjusted *p* > 0.05). No baseline marker was significantly associated with the PHQ-9 total score at follow-up (all *p* ≥ 0.138). The lowest nominal *p*-value was observed for KA (β = –0.55, *p* = 0.138), and after Hochberg correction all *p*(Hochberg) ≥ 0.550.

Similarly, to explore whether any associations were specific to the not at risk depression group, separate multivariate regression analyses were conducted within this group using both baseline and follow-up continuous depression scores. For baseline total depression scores, the strongest nominal association was for baseline suPAR (β = 0.367, *p* = 0.050); all other markers had *p* ≥ 0.98. However, the association did not remain significant after Hochberg correction. Using follow-up PHQ-9 scores as the outcome, none of the baseline markers showed evidence of association.

### Associations of Depression at Follow-up with Changes in Immune Marker Levels Over Time

From the overall cohort, follow-up immune marker data was only available for hsCRP (*n* = 40) and suPAR (*n* = 37). One participant who had hsCRP data did not have depression data, and all participants with suPAR data had depression data. We examined whether levels of these markers changed over the five-year follow-up period, using dependent t-tests. In the overall group, suPAR levels decreased significantly over the five-year follow-up period (3.8 (1.3) ng/mL vs. 2.8 (1.1) ng/mL, *p* < 0.001). No significant change in hsCRP levels was observed in (6.9 (12) mg/L vs. 11.2 mg/L *p* = 0.093) in the overall group.

Changes over time in suPAR and hsCRP levels were examined within the respective depression risk groups. In both the at risk for depression group (3.9 (1.2) ng/mL vs. 2.9 (1.1) ng/mL, *p* < 0.001) and the not at risk group (3.5 (1.0) ng/mL vs. 2.5 (1.0) ng/mL, *p* = 0.002), suPAR levels significantly decreased over the five-year follow-up period. In contrast, hsCRP levels did not show significant change in either the at risk for depression group (10.1 (14.8) mg/L vs. 16.3 (24.7) mg/L, *p* = 0.222) nor the not at risk group (2.56 (3.4) mg/L vs. 4.3 (4.6) mg/L, *p* = 0.103) over the five-year follow-up period.

We aimed to determine whether percentage change in inflammation over time was associated with the PHQ-9 total score at follow-up. Multivariate and logistic regression analyses, including both unadjusted and adjusted models, revealed no significant associations between the percentage change in immune marker levels (hsCRP and suPAR) and follow-up depression scores (continuous PHQ-9) or depression risk at follow-up.

### Relationship between Baseline Immune/Metabolite and Specific Depressive-Symptom Clusters/Subscales

When the PHQ-9 was partitioned into cognitive-affective and somatic subscales, distinct marker associations emerged. In adjusted linear models at baseline, the cognitive–affective subscale was not associated with any marker (all *p* ≥ 0.093; Hochberg-adjusted *p* > 0.05). In contrast, the PHQ-9 somatic subscale showed significant positive associations with QUIN (β = 0.57, *p* < 0.001), suPAR (β = 0.41, *p* = 0.001), Kyn (β = 0.46, *p* = 0.003), and hsCRP (β = 0.34, *p* = 0.007), and a negative association with the KA/QUIN ratio (β = −0.42, *p* = 0.004). These five findings remained significant after Hochberg correction for 11 tests.

In follow-up models, the 2015 cognitive-affective subscale was not associated with any baseline marker (smallest *p* = 0.052 for NGAL; all Hochberg-adjusted *p* > 0.05). The 2015 somatic subscale showed a nominal inverse association with baseline KA (β = −0.54, *p* = 0.025) and a borderline trend for the KA/QUIN ratio (β = −0.45, *p* = 0.057), but none remained significant after Hochberg correction.

Across all analyses, changes in inflammatory markers (ΔsuPAR or ΔCRP) between baseline and follow-up were not associated with 2015 PHQ-9 subscales (all *p* > 0.55).

## Discussion

This exploratory study in South African people living with HIV investigated the relationship between peripheral immune markers and Trp-Kyn metabolites and depression symptoms in a cohort of South Africans living with HIV over a five-year follow-up period. Our findings show that higher levels of baseline QUIN were positively associated with depressive symptom severity and increased likelihood for being at risk for depression at baseline. Further, lower baseline NGAL levels were associated with increased odds of having self-harm thoughts at follow-up. Baseline QUIN, Kyn, hsCRP, and suPAR were positively associated, whereas KA/QUIN was negatively associated, with somatic but not cognitive–affective depressive symptoms, indicating that inflammatory processes may predominantly influence the somatic dimension of depression in this cohort.

To clarify biological plausibility and aid interpretability, we outline a working pathway linking HIV infection to Trp-Kyn pathway dysregulation and depressive symptoms. HIV-1 proteins such as Tat and Vpr, together with chronic immune activation and elevated pro-inflammatory signalling (notably IFN-γ and IL-6), upregulate IDO1 and, to a lesser extent, TDO2, thereby shifting Trp metabolism toward Kyn (Samikkannu et al. 2009; Mondanelli et al. 2021). Monocyte and macrophage activation (indexed by sCD163) and broader immune activation (tracked by suPAR and hsCRP) further reinforce IDO1 activity and downstream flux (Shorer et al. 2024). Within this axis, Kyn is partitioned toward QUIN via microglial and monocyte pathways (Stone et al. 2022; Argolo et al. 2025), or toward KA via astrocytic Kyn aminotransferases (Parrott et al. 2016a). The resulting QUIN–KA balance reflects a neurotoxic (NMDA agonist) versus neuroprotective (NMDA antagonist) tone (Stone et al. 2022).

This model generates testable expectations that align with our findings. Higher QUIN and Kyn, together with lower KA/QUIN, should co-vary with somatic and vegetative symptom burden, such as fatigue, sleep, and appetite disturbances, that are more tightly linked to peripheral inflammatory signalling (Felger and Lotrich 2013; Majd et al. 2020). Consistent with this, we observed positive associations of QUIN, Kyn, suPAR, and hsCRP with the PHQ-9 somatic subscale, but no associations with the cognitive–affective subscale. If peripheral inflammation is a principal driver, broad inflammatory indices such as suPAR and hsCRP should track with somatic symptoms even when classical cytokines are variably detectable, as also observed previously (Foley, et al., 2021; Haupt et al. 2023). In a subtype C context, where Tat and Vpr sequence variants may amplify monocyte and microglial activation, this pathway would predict a shift toward QUIN, consistent with our baseline signal for QUIN and the inverse association with KA/QUIN (Campbell et al. 2007; Williams et al. 2020b; Asia et al. 2024a, b).

In addition to the Trp–Kyn findings, the association between lower baseline NGAL and increased odds of self-harm thoughts suggests that NGAL may capture a neuroimmune pathway related specifically to suicidality. NGAL is an acute-phase protein involved in neutrophil activation and neuroimmune signalling, and is upregulated in microglia and astrocytes during stress and inflammation where it affects synaptic plasticity and glutamatergic transmission (Ferreira et al. 2013). Stress-induced NGAL release has been shown to drive anxiety- and depressive-like behaviours in animal models (Yan et al. 2024), and elevated NGAL has been reported in individuals with depression and other neuroinflammatory psychiatric conditions (Mike et al., 2019; Raposo-Lima et al. 2021). However, NGAL responses are dynamic and context-dependent; blunted or low NGAL levels may reflect impaired or exhausted innate immune activation following earlier inflammation (Jha et al. 2015), a pattern described in some clinical subgroups including those with major depression disorder (Akter et al. 2023). This offers a plausible explanation for the inverse association in our cohort: lower NGAL may indicate a dysregulated or insufficient acute-phase response that marks individuals vulnerable to later self-harm thoughts. Given that NGAL emerged as the only biomarker in our study associated with suicidal ideation longitudinally, it may index a distinct neuroimmune phenotype in people with HIV and should be prioritized for further investigation.

Our results align with previous studies implicating the Trp-Kyn pathway in depression, particularly in the context of HIV-related neuroinflammation (Steiner et al. 2011; Keegan et al. 2016). In line with our findings, QUIN, a known neurotoxic metabolite (Drivsholm et al. 2021), was previously shown to associate with depression status (Drivsholm et al. 2021). Mechanistically, QUIN can drive excitotoxicity via N-methyl-D-aspartate (NMDA) receptor agonism, disrupting glutamatergic signalling and promoting synaptic injury (Lugo-Huitrón et al. 2013). Animal models show that stress-related depressive-like behaviour co-occurs with higher QUIN and glutamate and increased hippocampal NMDA subunit expression (Chen et al. 2013). In HIV, QUIN elevations have been linked to neurological complications in cerebrospinal fluid (CSF) (Valle et al. 2004). Elevated peripheral QUIN levels in individuals at risk for depression suggest that QUIN may be a marker involved in the mechanism of neuroinflammation and excitotoxicity underlying depressive symptoms in people with HIV. Studies also showed that cART improves depressive symptom severity partially by reversing disturbances in the Trp-Kyn pathway (Martinez et al. 2014). QUIN levels were shown to be 43% lower in sub-Saharan African individuals with HIV on cART in comparison to individuals with HIV who were treatment-naïve (Bipath et al. 2015). Somewhat similar, antidepressive treatment that reduced interferon-gamma induced depressive-like behaviour in rodents, decreased QUIN levels in the frontal cortex of these animals (Fischer et al. 2015). These findings support the hypothesis that HIV-associated depression has a biological basis linked to metabolic and immune dysregulation (Mudra Rakshasa-Loots 2023). As depression is common in this population, with rates substantially higher than in the general population, these findings emphasise the importance of identifying markers that could help to predict depression risk and guide clinical interventions. Importantly, the association between QUIN levels and depressive symptom severity and the fact that QUIN increased the odds for being at risk for depression in our study suggests that QUIN may not only serve as a marker of inflammation, as shown previously in a cohort South African people with HIV (Williams et al. 2025a), but should be investigated as a potential independent predictor of depression risk.

If replicated in larger cohorts, elevated QUIN, ideally as part of a parsimonious biomarker panel such as QUIN combined with the KA/QUIN ratio and a limited inflammatory marker, could support risk stratification in addition to standard symptom screening. A pragmatic workflow would retain universal PHQ-9 screening at routine HIV visits and reserve biomarker testing for research or tertiary-care settings when PHQ-9 scores are positive (for example, ≥ 10) or persistently subthreshold (for example, 5–9 across consecutive visits), or when clinical features suggest heightened neuroimmune activation. In such contexts, combined information from symptoms, biomarkers, and clinical factors could guide stepped-care that includes brief psychological interventions and close follow-up, escalation to pharmacological treatment and cART optimization where indicated, and, within trials, testing of anti-inflammatory or Trp-Kyn pathway-modulating strategies. Biomarkers should not be used as stand-alone diagnostic tests, and elevations may reflect comorbid inflammatory states such as intercurrent infections. Any implementation would require prospective validation demonstrating added predictive value beyond PHQ-9, assessment of confounding, clear clinical decision thresholds, turnaround times compatible with care, and cost-effectiveness analyses to support equitable adoption in South African health-system contexts.

Interestingly, we found that CRP, a key indicator of inflammation (Sproston and Ashworth 2018), did not change significantly over the 5-year follow-up period, suggesting that the inflammatory load may not have been substantially influenced by cART. While cART is highly effective in suppressing viral replication and improving immune function, it may not fully normalize systemic inflammation (Cai and Sereti 2021). Persistent low-grade inflammation, as reflected by stable CRP levels, has been reported in previous studies and is thought to contribute to long-term comorbidities in people living with HIV, including neurocognitive decline and depression (Pala et al. 2016). Given that peripheral Trp-Kyn pathway metabolites, including QUIN, can cross the blood–brain barrier (Skorobogatov et al. 2021) and are also produced by activated microglia in response to pro-inflammatory cytokines, the persistence of inflammation, even if not reflected by CRP, may continue to drive neurotoxic processes within the central nervous system. This notion is further supported by findings from preclinical studies, as it was showed that Trp-QUIN ratios remained elevated in the striatum of pigtailed macaques (a primate species) following cART treatment, suggesting persistent brain-specific activation of the Trp-Kyn pathway, despite normalized levels in the CSF. These results suggest that persistent activation of the Trp-Kyn pathway within the brain may contribute to HIV-associated depression, even in individuals who have achieved successful suppression of both peripheral and central nervous system viral replication with cART (Drewes et al. 2015). This supports the idea that localized neuroinflammation may persist and contribute to depression in HIV, even when systemic and CSF markers appear normal or remain unchanged after cART treatment.

## Limitations

Despite the valuable insights provided by this study, certain limitations must be acknowledged. First, the modest sample size and incomplete follow-up reduce statistical power and may have introduced selection bias. Participants who remained in the study might differ systematically from those lost to follow-up in ways that could affect associations between immune or metabolic markers and depression risk. Because metabolomics data were available only in a subset of participants, confidence intervals, particularly for QUIN, should be interpreted with caution due to small-sample variability. Moreover, as Trp–Kyn metabolites were quantified only at baseline, within-person change and temporal ordering cannot be assessed, leaving it unclear whether increases in QUIN precede or result from increases in depressive symptoms. Second, our study was limited to treatment-naïve individuals from a single geographic region, and thus, the generalisability of our results to broader South African and global populations with HIV requires further validation. Further, the absence of viral load data at baseline and follow-up limits our ability to adjust for viraemic burden, which can influence immune activation (including IDO1 activity) and Kyn metabolism. Although participants were treatment-naïve at baseline, and prior plasma sequencing in this cohort suggests detectable viremia, these observations cannot substitute for quantitative VL. Findings should therefore be interpreted cautiously, and future studies should incorporate VL to more precisely model inflammation–metabolite–symptom relationships. We employed the PHQ-9 as indicator of depression. Although it is a widely used and validated tool for assessing depressive symptoms, it functions as a screening rather than diagnostic instrument. A structured clinical interview conducted by a qualified professional like a clinical psychologist would have provided more accurate information on the presence or absence of major depressive disorder, symptom severity, and impact of the symptoms on the individual’s functioning. In addition, because repeated PHQ-9 assessments were limited, we could not evaluate trajectories or changes in depressive symptoms over time. Alternatively, administering the questionnaire at regular intervals would allow for tracking changes in the severity of individuals’ depression over time. The 5-year interval between marker assessment and follow-up depression evaluation warrants cautious interpretation. Numerous intervening psychosocial, health, and environmental factors could have influenced both marker profiles and mood outcomes during this period. However, we also not that BMI, smoking and alcohol consumption did not significantly differ between periods. Consequently, these findings should be viewed as exploratory associations rather than predictive models. Replication in studies with shorter follow-up intervals and repeated marker measures is needed to better delineate temporal dynamics.

## Conclusions

This exploratory study suggests that peripheral QUIN may be associated with depression risk and NGAL may be associated with self-harm thoughts in people living with HIV, warranting further investigation. Further, QUIN, Kyn, hsCRP, suPAR and KA/QUIN may be linked to somatic symptoms. Given the high prevalence of depression in individuals living with HIV, these results may be of value in identifying early markers for depression risk, which could aid in the development of targeted interventions to improve mental health outcomes. Further research is needed to confirm the causal relationship between QUIN, inflammation, and depression, and to explore the potential of inflammation based therapies in mitigating depression risk among people living with HIV.

## Data Availability

The datasets generated and/or analysed in the current study are available from the corresponding author on reasonable request and upon ethical approval.

## Declarations

### AI Declaration

During the preparation of this work the authors used Grammarly to assist with language editing/grammar. After using this tool, the authors reviewed and edited the content as needed and take full responsibility for the content of the publication.
